# Perspectives on Facilitators and Barriers to Successful Aging Among Latinos in the United States

**DOI:** 10.1093/geroni/igaf122.1383

**Published:** 2025-12-31

**Authors:** Phillip Cantu, Angela Gutierrez, Elizabeth Vásquez

## Abstract

Latinos are the fastest-growing segment of the older adult population in the U.S. Latinos’ reliance on informal care, well-documented barriers to health care access, and the continued immigration of older Latinos underscore the need for multifactorial, interdisciplinary approaches to address their unique aging trajectories. This symposium brings together perspectives from public health, sociology, occupational therapy, educational psychology, and health policy to examine psychosocial, caregiver, migration, and intervention factors contributing to healthy aging among Latinos. Researchers will highlight how health systems are innovating interventions to better serve Latinos, how caregiver-care recipient dyads’ stress are shaped by health conditions, the role of neighborhoods and loneliness in shaping cognitive trajectories, and how migration factors influence health outcomes. Taken together, these presentations will highlight strategies to improve health in late life for older Latinos while also accounting for unique barriers that are associated with adverse health outcomes. Since 2001, the International Conference on Aging in the Americas (ICAA) has been a driving force in advancing research on the experiences of aging Latinos in the U.S. and Mexico. What began as a primarily Southwestern U.S.-based network has since expanded into a national collective of senior researchers and emerging scholars. With research led by ICAA emerging scholars and RCMAR scientists, this session showcases diverse methodological approaches—qualitative research, quantitative analysis, and interventions—across various regional contexts in the U.S. to provide a comprehensive perspective on the facilitators, barriers, and policy recommendations for healthy aging at multiple ecological levels.

